# A Polygenic Score for Type 2 Diabetes Improves Risk Stratification Beyond Current Clinical Screening Factors in an Ancestrally Diverse Sample

**DOI:** 10.3389/fgene.2022.871260

**Published:** 2022-04-26

**Authors:** James R. Ashenhurst, Olga V. Sazonova, Olivia Svrchek, Stacey Detweiler, Ryosuke Kita, Liz Babalola, Matthew McIntyre, Stella Aslibekyan, Pierre Fontanillas, Suyash Shringarpure, 23andMe Research Team, Jeffrey D. Pollard, Bertram L. Koelsch

**Affiliations:** 23andMe, Inc., Sunnyvale, CA, United States

**Keywords:** polygenic score, type 2 diabees, consumer genomics, genetic risk, diabetes screening

## Abstract

A substantial proportion of the adult United States population with type 2 diabetes (T2D) are undiagnosed, calling into question the comprehensiveness of current screening practices, which primarily rely on age, family history, and body mass index (BMI). We hypothesized that a polygenic score (PGS) may serve as a complementary tool to identify high-risk individuals. The T2D polygenic score maintained predictive utility after adjusting for family history and combining genetics with family history led to even more improved disease risk prediction. We observed that the PGS was meaningfully related to age of onset with implications for screening practices: there was a linear and statistically significant relationship between the PGS and T2D onset (−1.3 years per standard deviation of the PGS). Evaluation of U.S. Preventive Task Force and a simplified version of American Diabetes Association screening guidelines showed that addition of a screening criterion for those above the 90th percentile of the PGS provided a small increase the sensitivity of the screening algorithm. Among T2D-negative individuals, the T2D PGS was associated with prediabetes, where each standard deviation increase of the PGS was associated with a 23% increase in the odds of prediabetes diagnosis. Additionally, each standard deviation increase in the PGS corresponded to a 43% increase in the odds of incident T2D at one-year follow-up. Using complications and forms of clinical intervention (i.e., lifestyle modification, metformin treatment, or insulin treatment) as proxies for advanced illness we also found statistically significant associations between the T2D PGS and insulin treatment and diabetic neuropathy. Importantly, we were able to replicate many findings in a Hispanic/Latino cohort from our database, highlighting the value of the T2D PGS as a clinical tool for individuals with ancestry other than European. In this group, the T2D PGS provided additional disease risk information beyond that offered by traditional screening methodologies. The T2D PGS also had predictive value for the age of onset and for prediabetes among T2D-negative Hispanic/Latino participants. These findings strengthen the notion that a T2D PGS could play a role in the clinical setting across multiple ancestries, potentially improving T2D screening practices, risk stratification, and disease management.

## 1 Introduction

The United States and other Western countries face an epidemic of type 2 diabetes mellitus (T2D). Population-wide screening is critical for identifying T2D-positive and prediabetic individuals in order to prevent severe pathology associated with more severe or protracted disease. Despite detailed screening guidelines developed by The U.S. Preventive Services Task Force and the American Diabetes Association (ADA), diagnostic delay in prediabetes and T2D continues to hamper timely and effective treatment (Samuels et al., 2006). In 2020, the Centers for Disease Control (CDC) estimated that over 7 million undiagnosed T2D cases exist among current U.S. residents, and a diagnostic rate of only 15.3% for the 80 + million individuals living with prediabetes (Centers for Disease Control and Prevention, 2020). By 2050, the number of undiagnosed cases could be over 13 million, as T2D prevalence is projected to increase to 25–28% of the U.S. population (Boyle et al., 2010).

This high rate of progression can be mitigated with improved screening and risk stratification methods. The T2D epidemic described above is not only a case identification problem but a resource allocation problem. Novel methods are needed to improve screening and risk stratification in order to most effectively allocate resources to healthcare providers managing the prevention and treatment of the disease.

The heritability of T2D has been estimated at 25–72% (Almgren et al., 2011; Florez et al., 2018), and genome-wide association studies (GWAS) have shown a highly polygenic architecture to be associated with risk for the disease (Xue et al., 2018). Thus, predictive genetic models that produce a polygenic score (PGS) containing many thousands of genetic variants have been increasingly investigated (Reisberg et al., 2017; Khera et al., 2018). Indeed, systematic reviews and an online depository of PGS together provide information about dozens of published distinct PGS for T2D, comprised of only three variants, to nearly 7 million variants (Padilla-Martínez et al., 2020; Lambert et al., 2021).

We hypothesized that a T2D PGS developed from a large-scale database and consisting of over 11,000 T2D-associated genetic variants would complement existing screening methods and improve individuals’ stratification across the T2D risk spectrum. First, we developed a novel PGS derived from a very large multi-ancestry sample in the 23andMe database; the PGS under study in this manuscript is not the one included in the 23andMe Personal Genome Service as of March 2022. Next, we hypothesized that the PGS would add unique predictive value over and above traditional factors that inform T2D screening decisions in the clinic: family history, age, and body mass index (BMI; Pippitt et al., 2016; American Diabetes Association, 2018; USPSTF, 2021). We also hypothesized that the T2D PGS would be associated with earlier age of onset of T2D, prevalence of prediabetes among those without a T2D diagnosis, T2D incidence after one year, and manifestations of severity including differences in T2D treatments and complications of T2D. Finally, given that PGS derived from samples of primarily European descent have exhibited limited transferability when assessed in other populations (Martin et al., 2019), we evaluated the T2D PGS in a second 23andMe cohort consisting of individuals with Hispanic/Latino ancestry to underscore the value of the T2D PGS as a clinical tool applied to those with ancestry other than European.

## 2 Materials and Methods

### 2.1 Study Participants and Survey Methodology

We recruited study participants from all genotyped 23andMe customers who opted to participate in research with 23andMe. All participants provided informed consent under a protocol approved by the external AAHRPP-accredited IRB, Ethical & Independent Review Services. Individual-level data from this study are not publicly available per the IRB-approved study protocol. Participants were included in the analysis on the basis of consent status as checked at the time data analyses were initiated.

A series of questions asked if a participant had ever been diagnosed with T2D by a physician. Those who answered affirmatively were considered cases, whereas those who indicated no personal history of T2D were considered controls. Participants who reported latent autoimmune diabetes in adults (LADA), maturity onset diabetes of the young (MODY), or only history of gestational diabetes were not counted as T2D cases. Participants without history of T2D diagnosis who reported any history of diagnosis of “high blood sugar or prediabetes” were counted as cases of prediabetes.

Those who reported a history of T2D diagnosis were asked follow-up questions about history of prescription treatment (metformin, insulin) and physician-directed lifestyle modifications. These participants were also asked about history of diagnosis of diabetes microvascular complications: neuropathy, nephropathy, and retinopathy.

Follow up surveys were made available one year later to ascertain if any participants had received a new diagnosis of T2D in the past 12 months. Incident cases were defined as those who had no existing diagnosis of type 2 diabetes at the baseline measurement at the time of enrollment, but who indicated a new diagnosis that occurred at least one but no more than two years after the initial question was answered. Additional questions asked about age of diagnosis of T2D, height and weight, and birth year. Ancestry category (European, Hispanic/Latino) was self-reported. Participants were required to have a minimum age of 20 and maximum age of 79 years old. Additional exclusions were: providing conceptually inconsistent responses like an age of T2D onset older than a currently reported age, reporting age of onset younger than age 20, reporting underweight or extreme obese BMI (BMI <18.5 or >69), or reporting a duration of time between initial diagnosis and current age greater than three standard deviations from the mean of this metric (>40 years). Individuals who were in the sample used for the GWAS or to train the PGS were excluded from the study.

**FIGURE 1:**
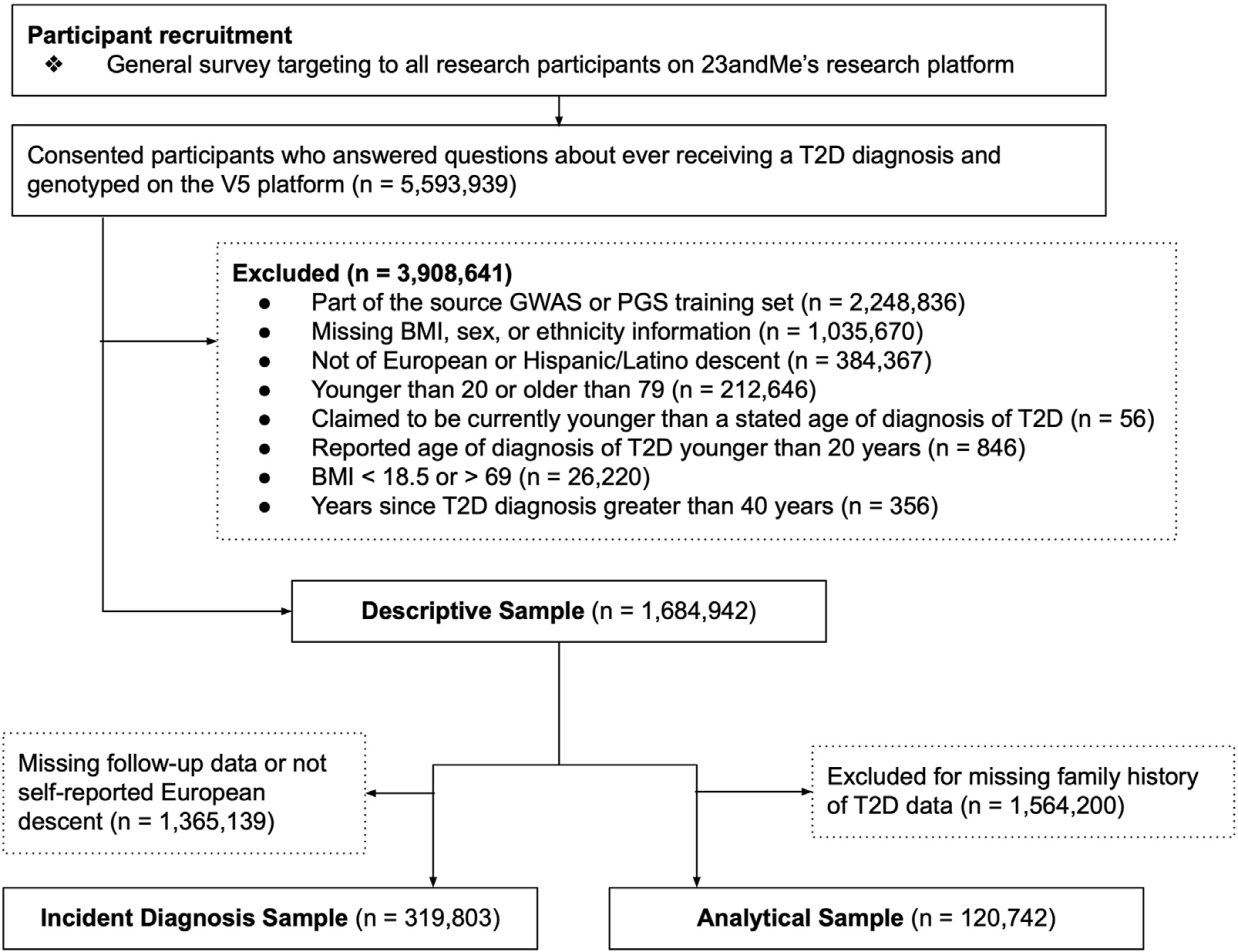
Participant recruitment and analysis flow diagram. Three data sets were used for components of this analysis. The Descriptive Sample was used to generate plots, to estimate raw prevalences, or to estimate unadjusted odds ratios. The Incident Diagnosis Sample was used to assess the association between the polygenic score (PGS) and incident diagnosis over time. The Analytical Sample was used for regression models that included family history as a predictor. Sub-sampling was required due to missing data in key survey questions required for analysis, and participant attrition over time.

Because a question from a separate survey was used to assess family history of T2D among first degree relatives, there were fewer available responses to this question relative to others, reflected in the participant flow diagram (Figure 1). To maximize sample size, descriptive analyses of the data (i.e., prevalence of T2D along the spectrum of the PGS) and unadjusted odds ratios between factors like the PGS and T2D prevalence include all available data (the Descriptive Sample), whereas regression analysis involving family history were performed in a subset of the full data set with family history data (Analytical Sample). Lastly, due to loss of participation with time, the sample used to assess incidence of T2D (Incidence Sample) also represents a subset of the full data, and there was only sufficient data to perform the analysis among those of self-reported European descent (Figure 1).

### 2.2 Genotyping and Polygenic Score Development

DNA extracted from saliva samples was assayed on the Illumina Infinium Global Screening Array (Illumina, San Diego, CA), consisting of approximately 640,000 common variants supplemented with ∼50,000 custom probes. This platform is referred to as 23andMe platform V5, and underwent quality controls as described previously (Nakka et al., 2019). Only participants genotyped on this platform are included in this analysis. A polygenic score associated with the likelihood of having T2D was developed using the methods described in 23andMe White Paper 23–21 (Ashenhurst et al., 2020). In brief, single nucleotide polymorphisms (SNPs) were selected from a meta-analysis of three GWAS conducted in individuals of European, Black/African American, and Hispanic/Latino descent. Candidate models based on nine variant sets determined by varying *p*-value and window distances were evaluated in tuning sets that were not included in the GWAS. Finally, based on best performance in the tuning cohorts, one variant set was chosen for final assessment in the European and Hispanic/Latino test cohorts, which were not included in the GWAS or model training.

The final model containing 11,999 SNPs showed a significant association with the likelihood of having T2D among participants of European descent [area under the receiver operator curve, AUC = 0.656, CI (0.654,0.659), Supplementary Table S2] as well as Hispanic/Latino individuals [AUC = 0.635, CI (0.628,0.642)]. Age and sex variables provided more information than the PGS alone in both the European descent [AUC = 0.774, CI (0.773,0.776)] and Hispanic/Latino [AUC = 0.811, CI (0.806,0.816)] subsamples. The combined model with demographic features and the PGS were the most predictive [European AUC = 0.814, CI (0.812,0.816), Hispanic/Latino AUC = 0.841, CI (0.837,0.845)]. The discriminative performance of this model ranks it among the leading models cited in the PGS Catalog as of March 2022 (Lambert et al., 2021). For complete detail about the PGS, see information in Supplemental Materials.

### 2.3 Statistical Analyses

Statistical analyses were conducted in statsmodels (v0.12.1) in Python (Seabold and Perktold, 2010). A study-wise significance threshold was defined as *p* < 0.0018 based on 28 independent comparisons and a Bonferroni correction. Reported odds ratios and linear model betas are adjusted for age, BMI (log transformed and standardized), sex, and first-degree relative family history of T2D unless otherwise described. All confidence intervals (CIs) provided are 95% CIs. To maintain participant privacy, counts or statistics that could uniquely identify fewer than five people are not provided in this manuscript.

## 3 Results

### 3.1 Participant Characteristics

The final Descriptive Analysis sample consisted of N = 1,528,668 individuals of European descent and N = 156,4274 of Hispanic/Latino descent. The subsample with available family history data (the European Analytical Sample, N = 113,126, Hispanic/Latino N = 7,616) was smaller, as was the sample with available repeated measures (European Incidence Sample, N = 319,803). Full sample descriptives are provided in Table 1, and participant exclusions are shown with a flowchart in Figure 1. The prevalence of self-reported T2D within each sex and decade of age in the multi-ancestry sample used to train the PGS are shown in Supplementary Figure S1. The median age of T2D diagnosis was 50 (mean = 48.3, SD = 11.2), and 43 (mean = 42.9, SD = 11.4) in the European-descent and Latino sub-samples, respectively.

**TABLE 1:** Sample descriptives.

Self-reported Ancestry	N	Age mean (SD)	Sex (%) (Female)	T2D Prevalence (%)
European	1,528,668	47.6 (15.8)	60.4	3.2
Hispanic/Latino	156,274	41.0 (14.2)	60.6	2.6
European sub-sample with family history data	113,126	53.3 (15.8)	66.5	4.6
Hispanic/Latino subsample with family history data	7,616	45.2 (14.9)	64.2	3.7
European sub-sample with one-year incidence data	319,803	50.5 (16.0)	68.3	0.9

The incidence sub-sample was composed of those who were T2D-negative at baseline and provided one year follow-up data.

### 3.2 The Polygenic Score Provides Information Not Captured by Family History

Current clinical practices rely heavily on family history of disease (FH) to identify patients at increased risk of developing conditions. But the full scope of heritability cannot be captured by FH alone, and not all individuals know their family history (e.g., those who were adopted), leaving open the possibility of under-identifying disease risk. We hypothesized that the T2D PGS combined with FH would improve the prediction of disease development more than either factor alone. This analysis was performed in the Analytical Sample (Figure 1).

**FIGURE 2:**
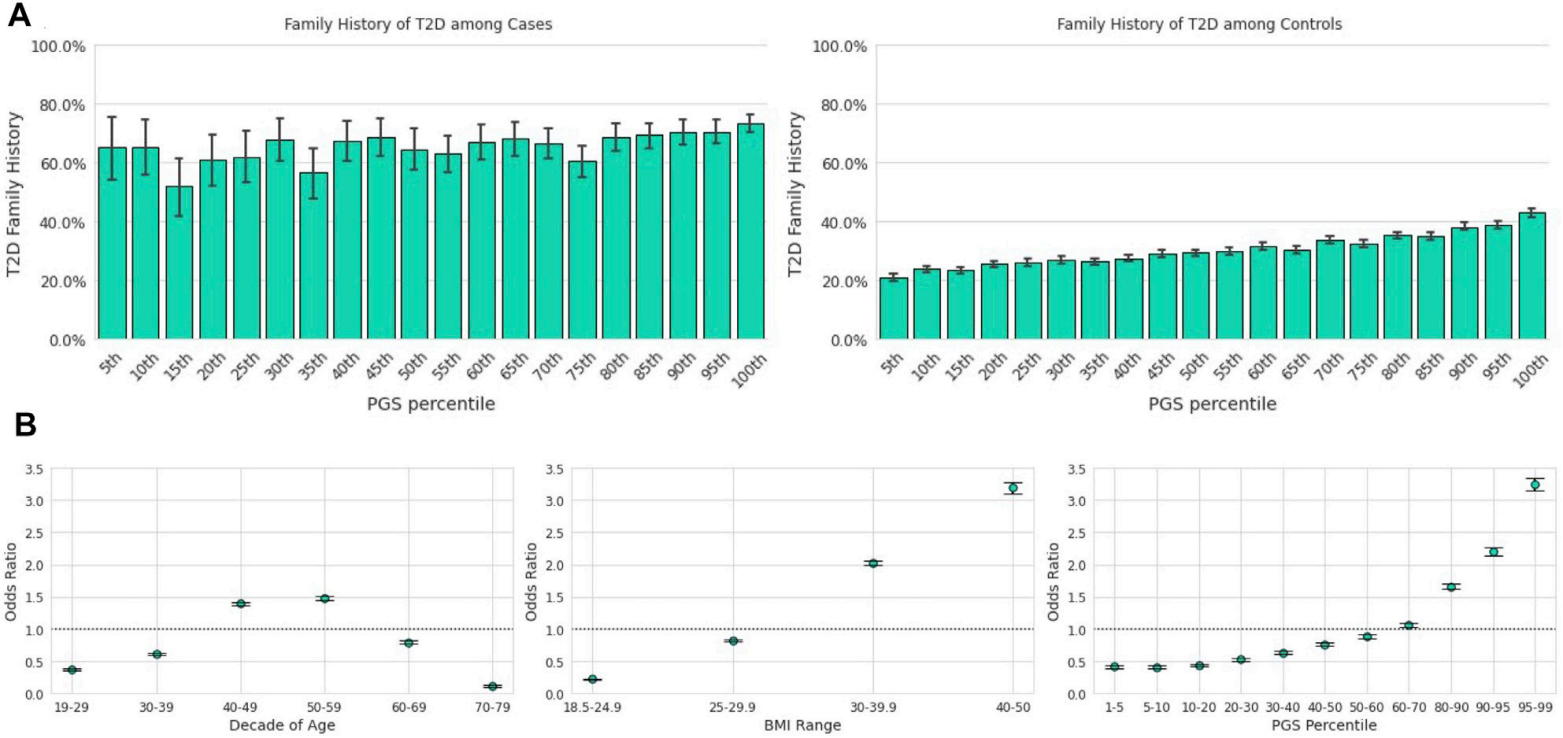
The T2D PGS is a predictor on par with traditional risk factors. Error bars here represent empirically derived 95% confidence intervals. **(A)**: Research participants who self-reported their family history were binarized into two groups: those with a first-degree relative with T2D and those without. The fraction of participants with a positive family history of T2D (y-axis) is plotted as a function of PGS ventile (x-axis) among T2D cases (left panel) and T2D controls (right panel). **(B)**: Unadjusted odds ratios (y-axis) of having T2D relative to the entire study population were calculated for each decade of age (left panel), BMI category (center panel), and PGS percentile (right panel). Error bars represent analytically computed 95% confidence intervals.

Among those in the lowest genetic risk ventile, 20.8% of controls and 65.2% of cases reported positive FH. Among those in the highest risk ventile, positive FH prevalence was 42.9% for controls and 73.1% for cases (Figure 2A). There was a significant relationship between family history status and the PGS across the Analytical Sample as estimated in a logistic regression model; each standard deviation in the PGS was associated with 32% greater odds of reporting family history of the condition [β = 0.27, *p* < 0.0018, OR = 1.32, CI (1.30,1.33)]

We next assessed several logistic regression models of T2D diagnosis as a function of the T2D PGS, positive FH, and the common T2D screening factors of age and BMI (Pippitt et al., 2016; Zheng et al., 2018) in a training sample, comprised of 75% of the analytic sample; a test set of 25% was reserved for model evaluation. Both FH and the PGS were statistically significant as predictors in separate models (Table 2) as well as in a model including both FH and PGS as predictors. The combined model had the best predictive performance [as assessed by Cox-Snell’s pseudo R2 statistic = 0.21, and by AUC in the out-of-sample test set, AUC = 0.85 (0.85,0.86)], compared to models with only FH [R2 = 0.19, AUC = 0.83 (0.83,0.84)] or only the PGS [R2 = 0.17, AUC = 0.83 (0.82,0.84)], showing that FH and PGS contribute unique information as predictors in each other’s presence.

**TABLE 2:** Logistic regression between prevalent T2D, family history, and the PGS among those of European descent.

Model	Base model	Family history only	Polygenic score only	Combined model
Intercept	−6.34	−6.82	−6.7	−7.13
Family History	-	1.34	-	1.23
Standardized Polygenic Score	-	-	0.48	0.43
Female Sex	−0.46	−0.57	−0.50	−0.60
Decade of Age	0.06	0.05	0.07	0.06
Standardized Log Body Mass Index	0.72	0.66	0.68	0.64
Cox-Snell’s Pseudo R2	0.14	0.18	0.18	0.21
Test Set AUC (95% CIs)	0.80 (0.79, 0.80)	0.83 (0.83, 0.84)	0.83 (0.82, 0.84)	0.85 (0.85, 0.86)

All coefficients derived from logistic regression were significant *p* <0.0018 in all models. N = 84,844 for all models. The model that included both family history and the PGS was the most predictive in terms of both pseudo R2 and out-of-sample AUC.

### 3.3 Potential Contribution of the Polygenic Score to Screening Practices

Although individual health care systems may use their own criteria, current screening guidelines often use two main sources: The U.S. Preventive Services Task Force (USPSTF, 2021) and the American Diabetes Association (ADA, 2018). The USPSTF currently recommends screening for abnormal blood glucose and T2D in adults 35–70 years of age who are overweight or obese and repeating blood glucose testing every 3 years if results are typical. Individuals from populations with higher prevalence of diabetes (American Indian/Alaska Native, Black, Hawaiian/Pacific Islander, Hispanic/Latino) should be considered for earlier screening (USPSTF). The ADA proposes screening for T2D beginning at age 45 for all people. Screening for prediabetes and onset of future T2D in asymptomatic people should be considered in adults of any age who are overweight and have one or more additional risk factors for diabetes (ADA). These risk factors include overweight and obesity, physical inactivity, abnormal lipid levels, high blood pressure, and smoking. Despite both screening recommendations, many at-risk individuals, as well as prediabetic and T2D cases, are being missed annually. We hypothesized that the T2D PGS could identify individuals who would benefit from earlier screening for T2D solely based on their genetic risk.

#### 3.3.1 Univariate and Multivariate Associations Between T2D Prevalence and Screening Factors

Using the T2D PGS in the Descriptive Sample, we calculated the unadjusted odds ratio (OR) of having T2D for a given PGS percentile range relative to the total population. We compared this outcome to the OR of the risk factors highlighted in both guidelines, age and BMI (Figure 2B), which were also calculated relative to the total study population. Age was scored as age of diagnosis for cases, and current age for controls. We observed substantial overlap in the unadjusted OR magnitudes associated with the three variables: The range of risk associated with the PGS, OR = 0.41 [CI (0.38,0.44)] at the 1st-5th percentile to OR = 3.25 [CI (3.16,3.35)] at the 95th-99th percentile, was comparable to the range associated with BMI, OR = 0.22 [CI (0.21,0.23)] at BMI 18.5–24.9 to OR = 3.19 [CI (3.11,3.28)] at BMI 40–50. Risk of prevalent T2D was highest for ages 50–59 [OR = 1.49, CI (1.45,1.52)] and lowest for ages 70–79 [OR = 0.11, CI (0.10,0.12)].

Age, BMI, and the PGS were statistically significant and independent predictors of T2D prevalence in a multivariate logistic regression model described in the prior section comparing competing models (Table 2). The jointly estimated odds were as follows: decade of age [OR = 1.07, CI (1.06,1.07)], log-transformed standardized BMI [OR = 1.90, CI (1.84,1.96)], and the standardized PGS [OR = 1.54, CI (1.50,1.58)], all ps <0.0018.

#### 3.3.2 Adding the Polygenic Score to Screening Guidelines

Another way to understand the utility of the application of specific screening guidelines is to estimate the sensitivity and specificity of those decision trees. We evaluated the application of USPSTF and ADA guidelines in our data with and without including the PGS in screening decisions. For these analyses, age of diagnosis was used for cases, and current age for controls. Hypothetical updated guidelines divide the PGS at those at or above the 90th percentile, versus those below.

The USPSTF criteria focus primarily on age and BMI. In our sample, the sensitivity of those criteria was 0.79, and the specificity was 0.58. To the USPSTF we added an additional criterion to screen individuals who are 35 or older, have normal BMI, but have a PGS at or greater than the 90th percentile. This resulted in an incremental increase in sensitivity (0.81) as well as a small decrease is specificity (0.56).

The ADA criteria include risk factors beyond the scope of this analysis (e.g., physical inactivity, history of cardiovascular disease, women with polycystic ovary syndrome, etc. (ADA, 2018). We chose to evaluate a simpler model that includes only age, BMI, and family history of T2D. Here, given the liberal criterion of screening all individuals 45 or older, the sensitivity was high (0.96) and the specificity was low (0.30). We added the additional criterion to screen adults (age 18 or older) with normal range BMI who have a PGS at or greater than the 90th percentile. This addition provided a small increase to sensitivity (0.97) and a slight decrease in specificity (0.28).

### 3.4 The Polygenic Score is Associated With Age of Diagnosis

**FIGURE 3:**
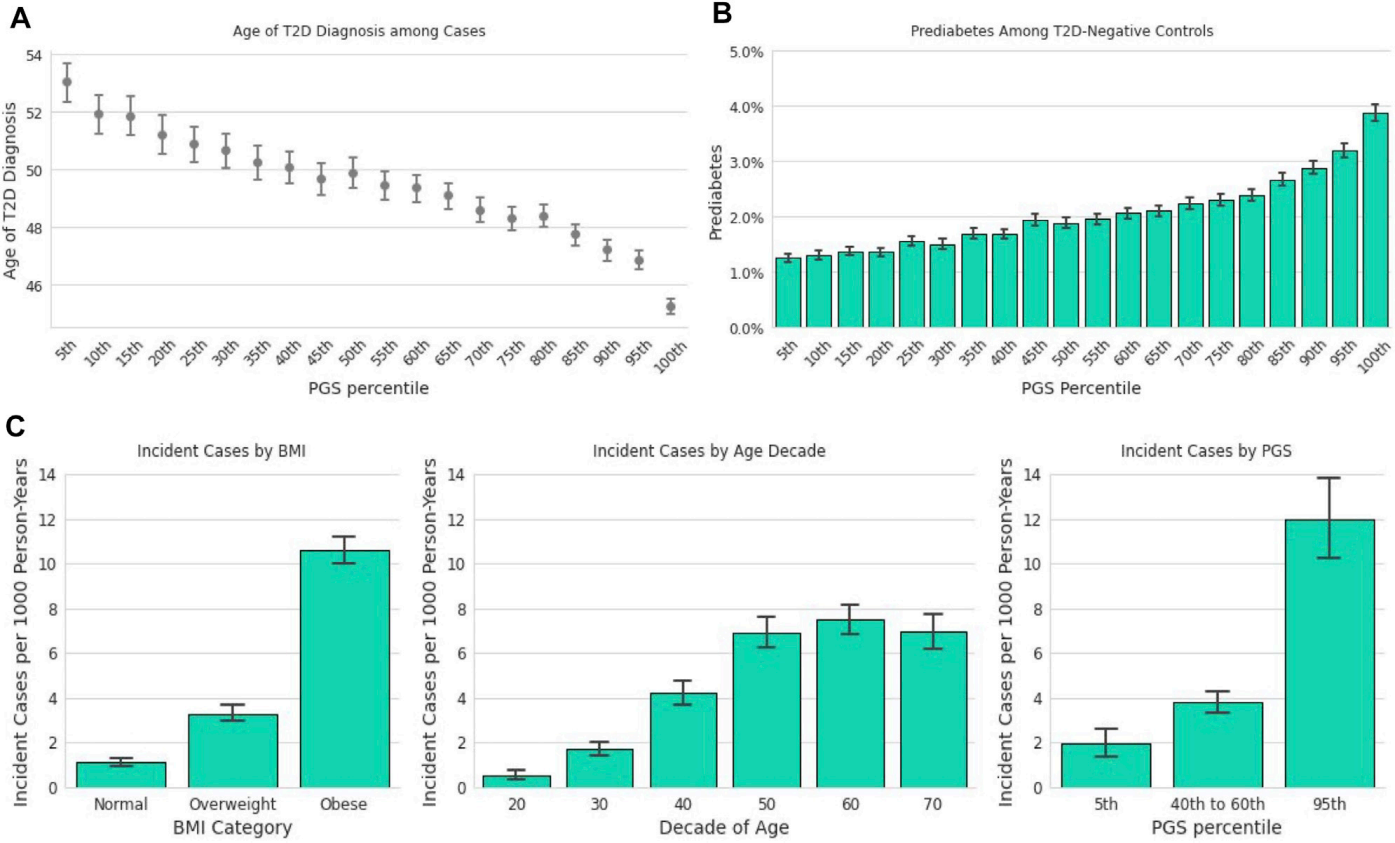
The T2D PGS is associated with diagnosis and incidence. **(A)**: Mean age at T2D diagnosis (y-axis) is plotted against PGS ventiles (x-axis) among participants who self-reported their age at T2D diagnosis. **(B)**: Prevalence of prediabetes (y-axis) is plotted for T2D-negative participants against ventiles of the PGS. **(C)**: A one-year incidence ratio was calculated among participants who were T2D negative at an initial time point and filled out a 1-year follow-up survey. T2D incidence (y-axis) was found to increase with increasing BMI (x-axis, left panel), with age up to the 60s (x-axis, middle panel), and PGS percentile (x-axis, right panel).

Earlier age of disease onset has been correlated with genetic risk for various conditions (Seibert et al., 2018; Mars et al., 2020). We examined the relationship between the T2D PGS and self-reported T2D age of diagnosis (AOD) to assess how well the model predicts disease development timing. In the Descriptive Sample, individuals in the lowest ventile of the PGS reported a mean AOD of 53.0 years compared to 45.2 years for those in the highest ventile, a difference of 7.8 years (Figure 3A). Furthermore, the T2D PGS was a statistically significant predictor for T2D AOD in a linear regression model that included BMI and family history of T2D in a subset of Analytic Sample who were T2D-positive and reported age of diagnosis (N = 4,663). Each standard deviation increase in the PGS was associated with a 1.37-year decrease in AOD [CI (−1.60, −1.16), *p* <0.0018], a relationship similar to that of standardized log of BMI [β = −1.73, CI (−2.04, −1.43), *p* < 0.0018]. Positive family history of T2D was not a significant predictor of AOD [β = −1.06, CI (−1.71, −0.41), *p* = 0.001, total model R2 = 0.07].

### 3.5 Prediabetes in Type 2 Diabetes-Negative Individuals

We hypothesized that the PGS model could also be used to predict the risk of prediabetes among those who were T2D-negative. Stratified by the T2D PGS, the prevalence of prediabetes in the highest PGS ventile in the Descriptive Sample was over 3-times the prevalence in the lowest PGS ventile, 1.3 vs. 3.9%, respectively (Figure 3B). We evaluated a logistic regression model of prediabetes diagnosis using age, BMI, T2D family history, and the T2D PGS as predictors among T2D-negative individuals in the Analytic Sample (*n* = 107,923). Each standard deviation increase of the PGS was associated with a 23% increase in the odds of prediabetes diagnosis [OR = 1.23, CI (1.19, 1.26), *p* <0.0018]. Prediabetes was also strongly associated with standardized log of BMI, [OR = 1.60, CI (1.55, 1.65), *p* < 0.0018] and family history of T2D, [OR = 2.03, CI (1.89,2.18), *p* < 0.0018], but not with female sex [OR = 1.05, CI (0.97,1.13), *p* = 0.2].

### 3.6 Incident Cases

In the subset of data with responses to annual follow-up surveys (Figure 1; Incident Diagnosis Sample), the mean time difference between the baseline response and the follow-up response was 446 days (SD = 102 days). The overall one-year incidence proportion, 4.86 per 1,000 person-years, is lower than but comparable to the 6.9 per 1,000 person-years statistic reported by the CDC for 2018 (Centers for Disease Control and Prevention, 2020). The incidence in the 23andMe database increased with decade of age, BMI, and PGS (Figure 3C). Stratified by PGS, the one-year incidence of T2D in the highest genetic risk ventile was over six times that of individuals in the lowest ventile (1.97 vs. 11.97 cases per 1,000), and roughly three times of individuals in the 40th-60th percentile (3.80 vs. 11.97 cases per 1,000). This rate of incidence among those with the greatest genetic risk was higher than those with obese BMI (10.64 cases per 1,000 person-years).

We evaluated a logistic regression model with incident case status as the outcome and age, standardized log BMI, T2D family history, and the PGS as predictors. The PGS proved to be a statistically significant predictor, where each standard deviation increase in PGS corresponded to a 43% increase in the odds of T2D incidence [OR = 1.43, CI (1.33,1.53), *p* < 0.0018], which was about half the incident risk associated with family history [OR = 3.02, CI (2.41,3.78), *p* < 0.0018], but was comparable to BMI [OR = 1.82, CI (1.67,1.99), *p* < 0.0018].

### 3.7 The Polygenic Score Informs Disease Progression

**FIGURE 4:**
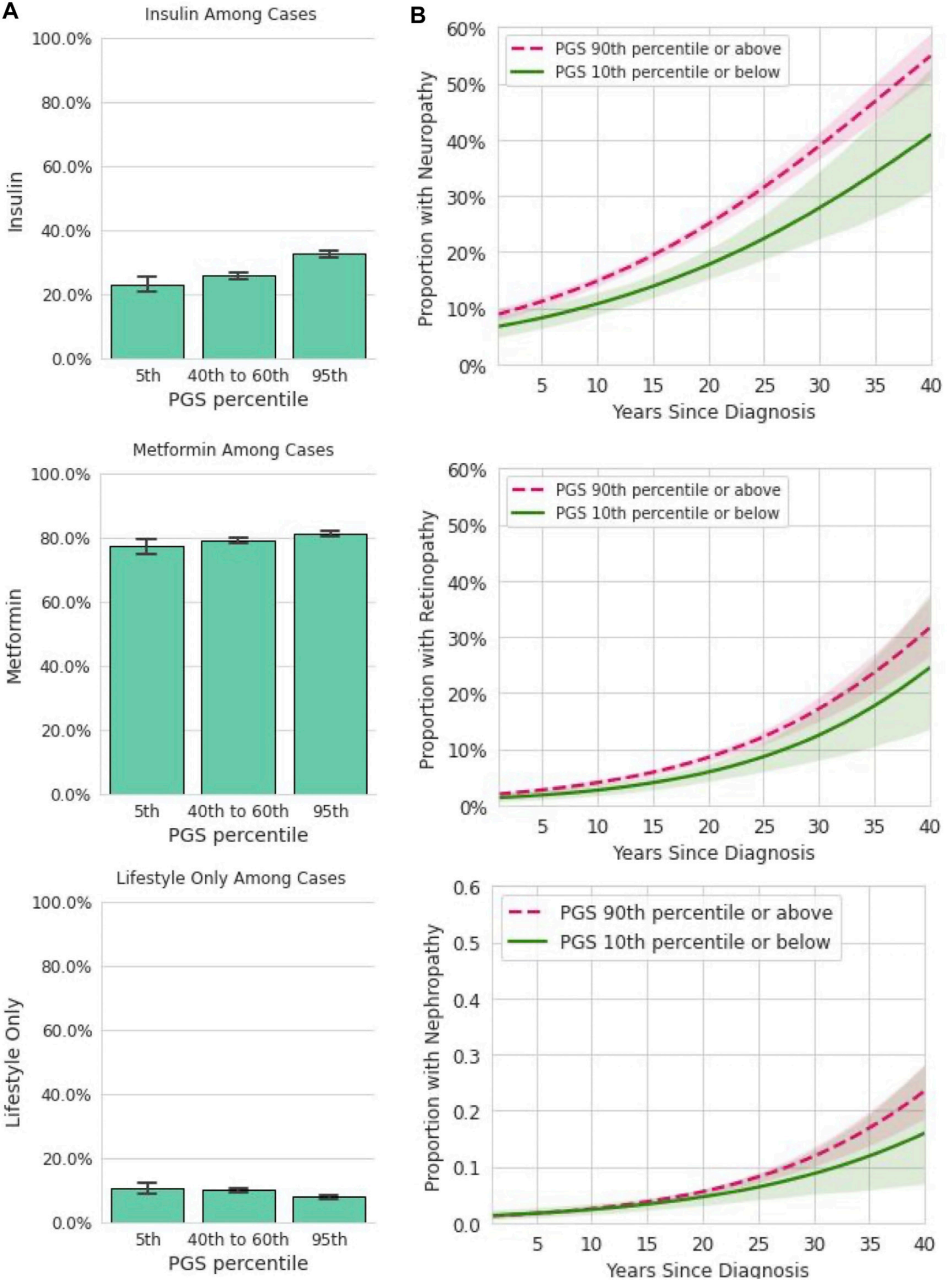
Among participants with T2D, the PGS is associated with some forms of treatment and disease complications. **(A)**: In a dataset restricted to participants who reported a T2D diagnosis and provided information on prescribed treatments, insulin, metformin, and lifestyle only are plotted (y-axis) for participants in the 5th, 40–60th, and 95th percentiles of the PGS (x-axis). Error bars represent empirically derived 95% confidence intervals. Insulin prescriptions were significantly associated with the PGS in multivariate models controlling for age, sex, BMI, and family history of T2D. **(B)**: Data shown are the relationship between years since T2D diagnosis and microvascular complications, stratified by PGS percentile in a logistic model. Shaded areas represent 95% confidence intervals. Neuropathy was significantly associated with the PGS in multivariate models controlling for age, sex, BMI, and family history of T2D.

We hypothesized that genetic risk for developing T2D as determined by the T2D PRS would also be associated with the risk of a more severe disease phenotype, as measured by the escalation of treatment strategy and by the rate of the development of T2D microvascular complications in a cohort of T2D-positive individuals in the Analytic Sample (Figure 1). We found that individuals with higher PGS values were more likely to be prescribed insulin (Figure 4A). We evaluated logistic regression models with the PGS, age, sex, and BMI to predict prevalence of prescribed treatment. Each standard deviation increase in the PGS was associated with 14% higher odds of being prescribed insulin [OR = 1.14, CI (1.09,1.19), *p* < 0.0018]. The PGS was not a statistically significant predictor of metformin treatment [OR = 1.05, CI (0.99,1.11), *p* = 0.09], or following only lifestyle modifications [OR = 0.89, CI (0.82,0.96), *p* = 0.004], Family history was significantly associated with metformin treatment [OR = 1.33, CI (1.14,1.55), *p* < 0.0018], but not insulin [OR = 1.21, CI (1.03,1.36), *p* = 0.02] or only lifestyle modifications [OR = 1.22, CI (1.00,1.48), *p* = 0.11].

We next assessed the utility of the PGS for predicting the rate of development of diabetes microvascular complications (Figure 4B). For this analysis, both current reported age and years since initial T2D diagnosis were entered into the logistic model in addition to the PGS, age, BMI, and sex. Each standard deviation increase in the PGS was associated with 10% higher odds of diabetic neuropathy [OR = 1.10, CI (1.04,1.16), *p* < 0.0018]. However, the PGS was not significantly associated with higher odds of diabetic nephropathy [OR = 1.05, CI (0.96,1.16), *p* = 0.25] or with diabetic retinopathy [OR = 1.07, CI (0.98,1.18), *p* = 0.12]. Family history was not associated with any of these three outcomes. Together, these data show the T2D PGS is associated with some but not all forms of disease severity as measured by prescribed treatment and prevalence of complications over time.

### 3.8 Polygenic Score Associations are Transferable to Hispanic/Latino Individuals

We hypothesized that the findings showing the relevance of the T2D PGS would replicate in other ethnicities. We were able to repeat many, but not all, of the specific analyses in the self-reported 23andMe Hispanic/Latino cohort (N = 156,410, see Methods and Materials and Figure 1 for participant recruitment flowchart).

**FIGURE 5:**
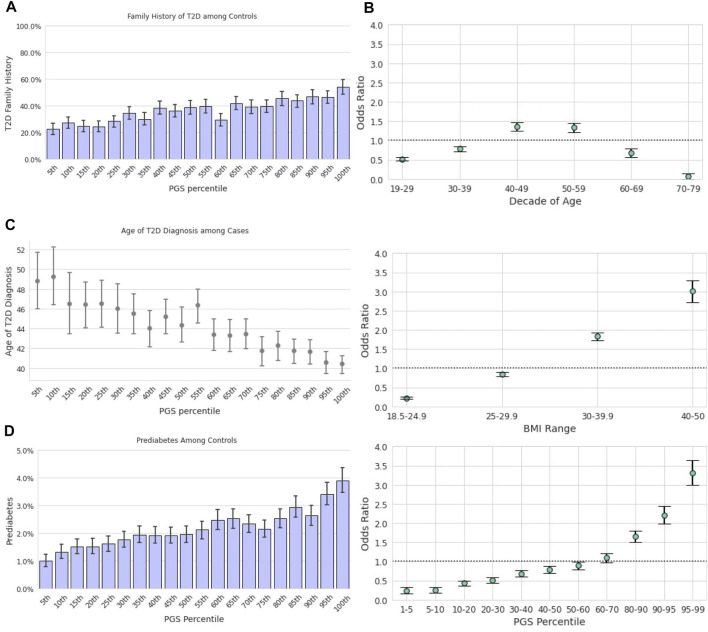
Repeated analysis in the Hispanic/Latino sample. **(A)**: The prevalence of family history of T2D among T2D-negative participants. Data among T2D-positive participants are not provided due to privacy practices. **(B)**: Odds ratios (y-axis) of having T2D relative to the Hispanic/Latino study population were calculated for each decade of age, BMI category, and Latino-specific PGS percentile. Error bars represent analytically computed 95% confidence intervals. **(C)**: Mean age at T2D diagnosis among cases (y-axis) is plotted against Hispanic/Latino-specific PGS ventiles (x-axis) among participants who self-reported their age at T2D diagnosis. Error bars represent empirically derived 95% confidence intervals. **(D)**: The prevalence of prediabetes among T2D-negative participants was significantly associated with the PGS, as shown with increasing ventiles of the PGS distribution. Data among T2D-positive participants are not provided due to privacy practices.

Among those who were T2D-negative at the time of the survey, family history of T2D was more common among those with higher genetic risk as indexed by the PGS than lower (Figure 5A; data for T2D-positive cases not shown due to smaller sample size and privacy requirements). As in the European-descent sample, family history was associated but not redundant with the PGS in a logistic model [OR = 1.42, CI (1.35,1.49), *p* <0.0018]. We examined the PGS performance as a predictor of T2D while controlling for T2D family history. This analysis showed the PGS to be a statistically significant predictor of T2D that provides unique information in a model containing age, BMI, family history, and the PGS [OR = 1.51, CI (1.37,1.67), *p* <0.0018; Table 3]. As in the sample of European descent, the model containing both the PGS and family history had the highest AUC in the Hispanic/Latino test set [AUC = 0.87, CI (0.85,0.91)].

**TABLE 3:** Logistic regression between prevalent T2D, family history, and the PGS in the Hispanic/Latino replication sample.

Model	Base model	Family history only	Polygenic score only	Combined model
Intercept	−5.82	−6.58	−6.15	−6.83
Family History	-	1.72	-	1.58
Standardized Polygenic Score	-	-	0.48	0.42
Female Sex	−0.46	−0.55	−0.48	−0.55
Decade of Age	0.06	0.05	0.06	0.05
Standardized Log Body Mass Index	0.74	0.69	0.70	0.67
Cox-Snell’s Pseudo R2	0.14	0.20	0.18	0.23
Test Set AUC (95% CIs)	0.83 (0.80, 0.87)	0.86 (0.83, 0.90)	0.86 (0.82, 0.90)	0.88 (0.85, 0.91)

All coefficients derived from logistic regression were significant *p* <0.0018 in all models. N = 5,712 for all models. The model that included both family history and the PGS, was the most predictive in terms of pseudo R2 and out-of-sample AUC.

We also examined the PGS’s ability to stratify Hispanic/Latino individuals by an unadjusted odds ratio of having T2D as compared to age and BMI (Figure 5B). Similar trends were observed as reported in the European cohort; the range of risk associated with the PGS, OR = 0.24 [CI (0.18,0.33)] at the 1st-5th percentile to OR = 3.32 [CI (3.02,3.64)] at the 95th-99th percentile, was comparable to the range associated with BMI, OR = 0.23 [CI (0.20,0.25)] at BMI 18.5 to 24.9 to OR = 3.01 [CI (2.75,3.29)] at BMI 40–50. Risk of prevalent T2D was highest for ages 40–49 [OR = 1.36, CI (1.26,1.47)] and lowest for ages 70–79 [OR = 0.07, CI (0.04,0.14)].

Addition of a hypothetical screening criterion at the 90th percentile of the PGS (as described in Section 3.3.2) to both the USPSTF and ADA criteria slightly increased sensitivity and reduced sensitivity. Our estimation of the sensitivity of USPSTF increased from 0.69 to 0.70 and reduced the specificity from 0.61 to 0.60. The addition to the simplified ADA criteria increased the sensitivity from 0.93 to 0.95, and decreased the specificity from 0.43 to 0.40.

We observed a correlation between increasing PGS and younger age of T2D diagnosis in the Hispanic/Latino cohort Figure 5C). Mean AOD ranged from 48.8 to 40.4 years from lowest to highest PGS ventile, a difference of 8.4 years. However, this relationship was not statistically significant [β = −0.61, CI (−1.62,0.40), *p* = 0.24] in a linear model trained to predict AOD from BMI, family history of T2D, and genetics in a small subset of the Hispanic/Latino cohort with complete data (N = 248).

Prediabetes in Hispanic/Latino T2D-negative participants was nearly four times more prevalent in those in the highest PGS ventile (3.9%) compared to the lowest ventile (1.0%; Figure 5D). We evaluated a logistic regression model of prediabetes diagnosis among T2D-negative individuals using age, BMI, T2D family history, and the T2D PGS as predictors. One standard deviation in the PGS was associated with a 36% increase in the odds of prediabetes among those without T2D [OR = 1.36, CI (1.22,1.51), *p* <0.0018], which was comparable to that of standardized log-BMI [OR = 1.64, CI (1.46,1.86), *p* <0.0018] and family history of T2D [OR = 1.60, CI (1.22,2.11), *p* < 0.0018].

Insufficient data were available in the Hispanic/Latino cohort to evaluate the association between the T2D PGS and incident diagnosis, treatment prevalences, or microvascular disease complications.

## 4 Discussion

Type 2 diabetes is a disease of metabolic dysregulation that begins years before symptoms are evident and complications arise. An estimated 1 in 3 American adults have prediabetes and 5–10% of these individuals will receive a T2D diagnosis within one year (Tabák et al., 2012). Lifestyle can be extremely successful in reversing the course of the disease, mostly when initiated early (Glechner et al., 2018). Thus, there is potential for polygenic scores to identify additional people who may be overlooked by traditional screening methods and who could benefit from earlier lifestyle modifications and medical intervention. Although the real-world impact of incorporating a T2D PGS in clinical practice remains to be thoroughly studied, we demonstrate its utility in identifying individuals with increased risk for prediabetes among the T2D-negative population. Furthermore, the PGS is also highly correlated with earlier age of T2D onset and can be used to predict incident T2D cases from a population of susceptible individuals. We also found the risk profile conferred by increasing PGS to be comparable to risk associated with increasing age and BMI. Taken together, these findings argue strongly for including a T2D PGS in a clinical assessment of T2D risk and prophylactic decision-making if available.

### 4.1 Incorporating Genetic Risk Into Screening Tools

Studies are beginning to hint at the clinical utility of PGS. Still, the combination of FH and PGS as a more robust method of predicting the individual likelihood of developing a complex disease has yet to be fully explored. Clinicians recognize that at-risk individuals may be missed when relying on FH alone for disease prediction and that gathering a FH is time-consuming and often neglected. Furthermore, not all individuals have knowledge of family history. A clinical tool encompassing FH and PGS may improve disease prediction.

Previous publications have employed several methods to assess whether polygenic scores add predictive utility when used jointly with family history, including examining predictive model performance (Sun et al., 2013; Helfand, 2016; Hughes et al., 2021) and determining whether risk estimates for PGS remained significant after adjustment for family history (Tada et al., 2016). In the present study, we observed an increasing relationship between both T2D genetic risk and positive family history among European-descent and Hispanic/Latino-descent T2D-negative individuals. We also found, however, that family history is associated with but not equivalent to genetic risk. Factors other than genetics, such as common environment, may also contribute to the risk conferred by family history, and polygenic inheritance results in more generational variability than monogenic patterns (i.e., Mendelian inheritance). Ultimately, a model including both family history and the PGS proved better at predicting T2D than each factor separately in terms of pseudo R2, out-of-sample AUC, and sensitivity when added to both USPSTF and ADA guidelines in both the European-descent and Hispanic/Latino cohorts. These results indicate that information captured in the PGS is not completely redundant with family history, and that disease risk is most comprehensively assessed when genetic analysis is combined with standard clinical risk factors.

Screening for prediabetes and T2D is often based on a set of guidelines that determine eligibility based on well-documented risk factors such as age, BMI, positive family history, membership in a high-risk race or ethnic group, and environmental or behavioral factors (Pippitt et al., 2016). In the present study, we have demonstrated the validity of the T2D PGS as a risk factor that contributes information over and above family history. Addition of the PGS to the USPSTF screening guidelines incrementally improved sensitivity, with a corresponding small decrease in specificity. We note that ADA guidelines, however, have very high sensitivity with or without the PGS.

Optimization of the sensitivity and specificity of these guidelines within medical systems could include the PGS as a risk factor, considering that it does provide some information that is independent of family history. It is beyond the scope of the present study, but medical economic analysis could find that screening younger people who may not have traditional risk factors but do have a higher PGS, and perhaps delaying screening for older people with no risk factors and a low PGS could balance sensitivity, specificity, and screening costs. This optimization is even more plausible as costs for genome-wide genotyping continue to decrease. Indeed, a single genomic assay could be used for multiple purposes beyond T2D screening throughout a person’s life.

### 4.2 Genetic Risk and Disease Severity

In addition to identifying more cases of T2D, several studies have suggested that genetic screening could be useful for predicting disease severity (Paul et al., 2018; Oetjens et al., 2019; Chen et al., 2020). T2D impacts individuals differently; some experience mild symptoms, controlled relatively easily by lifestyle intervention and minimal therapeutic intervention, while others experience severe complications and have a difficult time with disease management. Many patients progress from nonmedical, lifestyle-only treatment to medications like metformin, and some require insulin as their condition shifts from impaired glucose tolerance to insulin insufficiency. T2D severity is also closely associated with diabetic microvascular complications, the most common of which are diabetic retinopathy, nephropathy, and neuropathy.

In the present study, we found the T2D PGS to correlate with treatment options where those at higher PGS were more likely to be treated with insulin. Metformin treatment or lifestyle-only interventions were not significantly associated with the PGS. Yet for complications of T2D, the PGS was markedly related to the rate of neuropathy diagnosis, but not to nephropathy and retinopathy. Further work may identify sub-scales within a T2D PGS that associate with specific biological pathways or systems, illuminating specific causes of genetic risk and complications (Udler et al., 2018; Tremblay et al., 2021). Together, these findings are only an initial indication that the T2D PGS may be indicative of specific forms of disease progress, but further studies are needed to explore this thoroughly.

### 4.3 Assessing Genetic Risk in People of all Ancestral Backgrounds

Type 2 diabetes is on the rise across the world and in the United States its burden is disproportionately felt by Black/African Americans and Hispanic/Latino individuals (Centers for Disease Control and Prevention, 2020). Thus, the clinical utility of the T2D PGS is especially relevant for non-European individuals. Taken in the context of the massive Euro-centric bias in the field of polygenic risk prediction (Martin et al., 2019), we considered it important to evaluate the application of the PGS in a non-European population with a sufficient sample size for most of this analysis. It is critical that individuals from all backgrounds be provided the opportunity to participate in genomic research, and that all efforts are made to assess and calibrate PGS in diverse samples.

We selected the 23andMe Hispanic/Latino cohort because this T2D PGS has roughly comparable performance in this group as in European-descent individuals, as evidenced by the AUROC (0.656 in European-descent and 0.635 in Hispanic/Latino-descent individuals) and other risk stratification statistics, and because we had sufficient family history data in this cohort for a sufficiently powered study. Our analyses show that, as in the European cohort, the PGS provides valuable information for identifying at-risk Hispanic/Latino individuals, on par with risk factors already used for clinical decision-making. These findings serve as an important proof of principle for the application of polygenic prediction to assessing risk in underserved populations. 23andMe’s efforts to recruit a more diverse pool of study participants (23andMe, 2019) will enable additional follow-up studies with population-specific versions of the T2D PGS in order to deliver better value to our customers and provide more accurate tools for clinicians and their patients.

### 4.4 Limitations and Conclusions

The present study has several limitations that should be considered when interpreting the results. All phenotypes were obtained through participant self-report, although 23andMe’s previous work has shown the accuracy and robustness of this form of data collection at scale (Eriksson et al., 2010; Tung et al., 2011). We expect the additional granularity into treatments, disease complications, and biomarker/fasting glucose data obtained through clinical health records would likely improve the ability of the PGS to predict these phenotypes in T2D-positive individuals, as well as more precision in the definition of a participant with “prediabetes.” Missing data across survey instruments resulted in smaller subsamples used for regression modeling compared to the larger sample with T2D diagnostic and demographic information. Models assumed linear relationships between the outcomes and age or BMI, whereas non-linear relationships may better explain the data. Additionally, due to limited family history and incident data, we were unable to expand our analyses beyond those of European and Hispanic/Latino descent.

Typically, PGS (including this one) do not include rare variants with large effects, which, if present, would contribute far more risk than the polygenic background of common variants; nonetheless, being rare, most people do not carry these variants, and a PGS based on common variants would be relevant for most of the population. To maintain the scope of the present study, our evaluation of the sensitivity and specificity of the ADA guidelines did not include all risk factors included in the guidelines, and we did not attempt optimization of screening decision thresholds, including economic analyses. The analysis of microvascular complications of diabetes did not account for individual differences in treatment history, which would also affect the rate of development of these complications. We did not have data representing the age of onset of these complications, precluding survival analysis.

In this paper we present the possible clinical relevance of a T2D PGS as a predictor of disease risk and severity that provides some information that is independent of family history. Given this, the PGS could be considered as an additional risk factor in screening guidelines and could be used to help inform clinical decision making. The replication of many findings in a Hispanic/Latino cohort indicates the transferability across other populations when datasets of sufficient size exist and PGS with sufficient performance can be developed.

## Data Availability

Individual-level data from this study are not publicly available per the IRB-approved study protocol.
