# Supplementary material for: A Polygenic Score for Type 2 Diabetes Improves Risk Stratification Beyond Current Clinical Screening Factors in an Ancestrally Diverse Sample
Source: Front Genet. 2022 Apr 26;13:871260. doi: 10.3389/fgene.2022.871260 (PMC9086969; doi:10.3389/fgene.2022.871260)
Supplement: Supplementary file 1 [file DataSheet1.docx]

Supplementary Material

# Supplementary Methods

## Polygenic Score Development Methods

### Phenotype definition

Cases and controls were defined based on self-reported responses to questions about current or past diagnosis of type 2 diabetes. All question phrasings focused on receiving a diagnosis of or treatment for type 2 diabetes. Although some phrasings had minor differences, the general phrasing was: “Have you ever been diagnosed with, or treated for, type 2 diabetes”. Participants answering affirmatively for either condition were treated as cases, while those who report no history of type 2 diabetes diagnosis were counted as controls. Participants who reported latent autoimmune diabetes in adults (LADA), maturity onset diabetes of the young (MODY), or only history of gestational diabetes were not counted as T2D cases. Participants who reported any history of diagnosis of “high blood sugar or prediabetes” were counted as cases of prediabetes.

Those who reported a history of T2D diagnosis were asked follow-up questions about history of prescription treatment (metformin, insulin) and physician-directed lifestyle modifications. These participants were also asked about history of diagnosis of diabetes microvascular complications: neuropathy, nephropathy, and retinopathy.

### Model description

Model development was conducted as described previously (Ashenhurst et al., 2020). For the development of the polygenic score (PGS), there was sufficient data to attempt ancestry-specific GWAS among those of European, Hispanic/Latino and Black/African American descent. These three GWAS were combined in a meta-analysis, from which SNP sets were selected. The GWAS meta-analysis summary statistics were pruned using p-value thresholds: 0.05, 0.005, 0.0005, and window distances: 0, 10,000, 50,000 kb.

For model training, the European, Hispanic/Latino, and Black/African American training cohorts were combined into a mega-cohort, with genetic features optimized against ancestry-specific validation sets composed of individuals of European, Hispanic/Latino, Black/African American descent.

Two variant sets were selected as the best model based on performance in the tuning sets, and were finally assessed in ancestry-specific test sets (Supplemental Table 2). Additional features included in model training were age and age^2, interactions between sex and age terms, as well as the first ten global principal components (PCs) to account for population stratification.

# Supplementary Figures and Tables

## Supplementary Figures


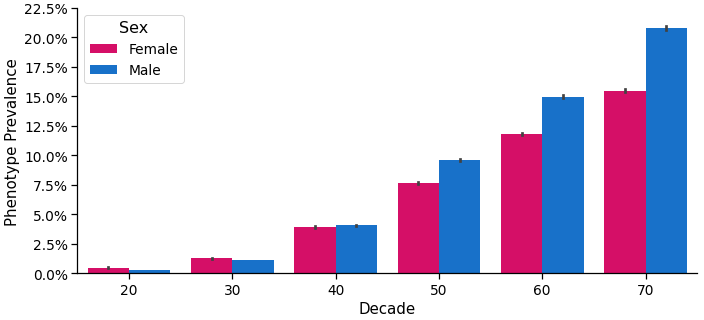
**Supplementary Figure 1**. T2D prevalence in the multi-ancestry polygenic score training cohort.


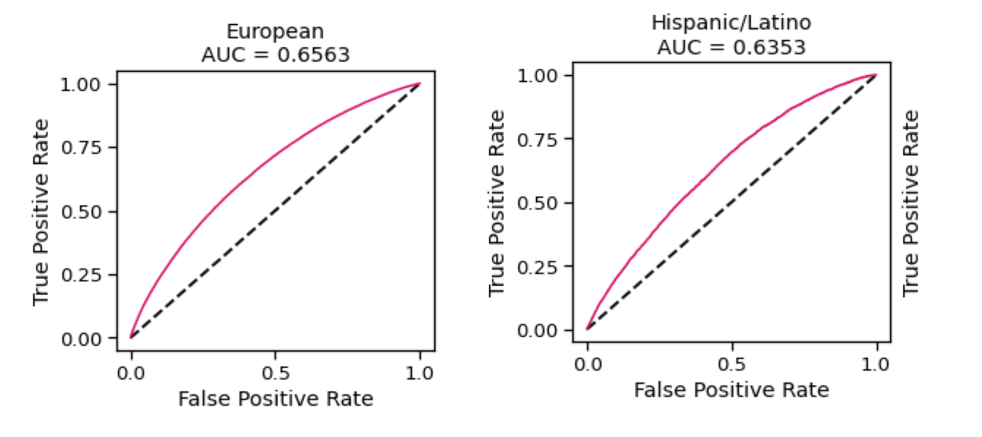


**Supplementary Figure 2**. Area under the receiver operator curve (AUC) in ancestry-specific test cohorts for the polygenic score. Demographic covariates age and sex were included in the full model, but were not included in these calculations of AUC.


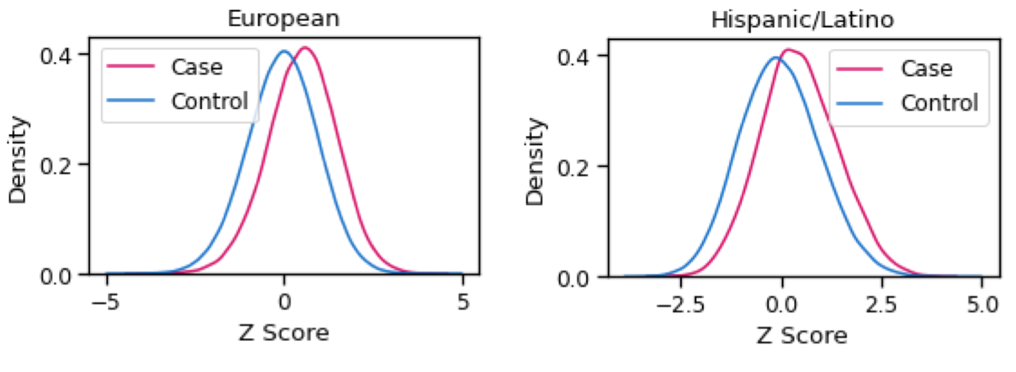


**Supplementary Figure 3**. Polygenic score distributions of cases and controls in ancestry-specific test sets


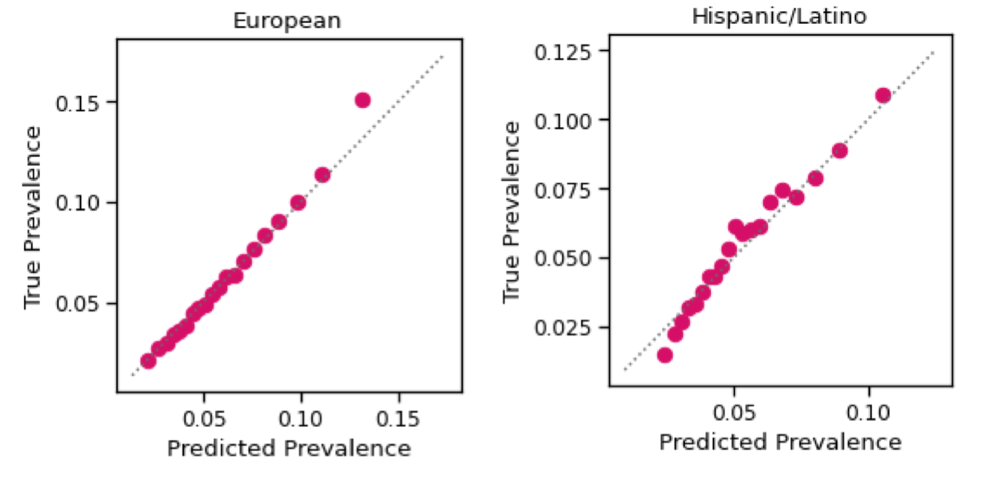


**Supplementary Figure 4**. Calibration plots comparing the observed and estimated phenotype prevalence per ventile of the polygenic score in ancestry-specific test sets.

## Supplementary Tables

### Polygenic score results

**Supplementary Table 1.** Polygenic score development participant descriptive statistics

| **Sample Use** | **Genotyping Platform** | **Ancestry group** | **N** | **Age mean (SD)** | **Sex (% female)** | **Phenotype prevalence (%)** |
| --- | --- | --- | --- | --- | --- | --- |
| GWAS | V1 to V5 | European | 1,680,210 | 48.2 (15.7) | 58.7% | 6.3% |
|  |  | Hispanic/Latino | 373,496 | 40.3 (13.8) | 59.1% | 5.5% |
|  |  | Sub-Saharan African/African American | 134,934 | 41.7 (14.4) | 60.1% | 7.4% |
|  |  | East/Southeast Asian | 60,530 | 37.9 (12.8) | 61.0% | 3.2% |
| Training multi-ancestral models | V5 | European, Hispanic/Latino, Sub-Saharan African/African American | 2,249,141 | 46.2 (15.7) | 58.9% | 6.2% |
| Testing | V5 | European | 720,750 | 48.2 (15.7) | 58.7% | 6.3% |
|  |  | Hispanic/Latino | 93,397 | 40.3 (13.8) | 58.7% | 5.5% |
|  |  | East/Southeast Asian | 60,422 | 37.9 (12.8) | 61.1% | 3.1% |
|  |  | South Asian | 32,616 | 39.8 (12.7) | 42.5% | 6.1% |
|  |  | Northern African/Western Asian | 26,542 | 42.3 (14.4) | 45.5% | 3.9% |
|  |  | Sub-Saharan African/African American | 15,065 | 41.7 (14.3) | 60.6% | 7.2% |

**Supplementary Table 2.** PGS model performance metrics

| **Ancestry group (test sets)** | **Full model AUROC (95%CIs)** | **Genetics only AUROC (95%CIs)** | **Odds ratio top 5% versus average (95% CIs)** | **Odds ratio top 5% versus bottom 5% (95% CIs)** |
| --- | --- | --- | --- | --- |
| European | 0.8139 (0.8124 to 0.8155) | 0.6563 (0.6539 to 0.6586) | 3.3 (3.15 to 3.39) | 9.5 (8.75 to 10.25) |
| Hispanic/Latino | 0.8405 (0.8365 to 0.8446) | 0.6353 (0.6282 to 0.6424) | 2.2 (2.0 to 2.49) | 10.3 (7.83 to 13.5) |

*Note*: "Full model" includes the features age, age^2^, sex, age*sex, age^2^, and the PGS. "Genetics only" consists of only the PGS. Genome-wide principal components, which were included in training, are not included in the evaluation of model performance.
